# Growth and lipid accumulation by different nutrients in the microalga *Chlamydomonas reinhardtii*

**DOI:** 10.1186/s13068-018-1041-z

**Published:** 2018-02-13

**Authors:** Lei Yang, Jun Chen, Shan Qin, Min Zeng, Yongguang Jiang, Lang Hu, Peng Xiao, Wenlong Hao, Zhangli Hu, Anping Lei, Jiangxin Wang

**Affiliations:** 1 Shenzhen Key Laboratory of Marine Bioresource & Eco-environmental Science, Shenzhen Engineering Laboratory for Marine Algal Biotechnology, Guangdong Provincial Key Laboratory for Plant Epigenetics, College of Life Sciences and Oceanography, Shenzhen University, Shenzhen, 518060 Guangdong People’s Republic of China; 2 Nanshan District Key Lab for Biopolymers and Safety Evaluation, College of Materials Science and Engineering, Shenzhen University, Shenzhen, 518060 Guangdong People’s Republic of China

**Keywords:** *Chlamydomonas reinhardtii*, Nutrient depletion, Sodium acetate, Lipid accumulation, Metabolomics

## Abstract

**Background:**

Individual nutrient depletion is widely used to induce lipid accumulation in microalgae, which also causes cell growth inhibition and decreases the total biomass. Thus, improving the lipid accumulation without biomass loss in the nutrient deficiency cells becomes a potential cost-effective treatment for cheaper biofuels.

**Methods:**

In this study, the effects of different nutritional conditions on the growth and contents of lipids in *Chlamydomonas reinhardtii* were compared, and the metabolic profiles under different nutritional conditions were also investigated.

**Results:**

We showed that similar to other microalgae, nitrogen or phosphorus deficiency inhibited the growth of *Chlamydomonas* and combined nutrition deficiency reduced biomass by up to 31.7%, though lipid contents in cells (g/g dry weight [DW]) were significantly increased. The addition of sodium acetate countered this growth inhibition that resulted from nitrogen and phosphorus deficiency, with significantly increased biomass. Furthermore, the combination of 4 g/L sodium acetate supplementation with nitrogen and phosphorous deficiency increased total fatty acid yield (mg/L) by 93.0 and 150.1% compared to nutrient-depleted and normal culture conditions, respectively. Metabolite content was affected by the different nutritional conditions, especially metabolites that are involved in lipid metabolism, amino acid metabolism and metabolism of external substances.

**Conclusion:**

Further research into these metabolites could shed light onto the relationship between cell growth inhibition and fatty acid accumulation in *Chlamydomonas*.

**Electronic supplementary material:**

The online version of this article (10.1186/s13068-018-1041-z) contains supplementary material, which is available to authorized users.

## Background

Renewable, environmental friendly biofuels is among the attractive research and development fields that are much needed because of fossil energy depletion and environmental pollution. The most utilized raw materials in bioenergy production are oil-producing crops such as soy, corn, rape and castor. However, the use of these food resources for energy production raises economic concerns [1–3]. Microalgae have high photosynthetic efficiency, growth rate, biological yield, and they do not use arable land, which makes algae a superior potential biofuel candidate [4]. The model green microalga, *Chlamydomonas reinhardtii*, is widely selected for biofuel experiments, because of its advantages, such as fast growth, short generation time, strong adaptability and easy cultivation [5].

Current research into increasing fatty acid (FA) accumulation in microalgae mainly focuses on screening microalgae species, optimization of culture conditions and transformation of microalgae by genetic engineering. A commonly used technique is to limit key nutrient elements, mainly nitrogen and phosphorus, which inhibits microalgae growth but promotes biosynthesis of FA. There is thus a discrepancy between reduced biomass (dry weight [DW] g/L) and increased lipid production (g/g DW), and high oil content is often achieved by sacrificing biomass [6]. The addition of organic carbon sources (such as glucose, ethanol, methanol, acetate) to the microalgal culture medium during cultivation can promote not only cell growth, but also lipid yield (g/L) therein. For instance, sodium acetate addition can increase the yield of intracellular FA in both *C. reinhardtii* [7] and another green microalga *Haematococcus pluvialis* [8]. Sodium acetate also promotes the growth of *Chlorella pyrenoidosa* and increases its lipid yield [9]. Therefore, it might be an effective method to improve the total yield of lipids while not reducing the biomass by heterotrophic or mixotrophic culture.

Metabolomics is about the measurement and study of the small-molecule metabolites that constitute biochemical networks and metabolomics has been widely applied in microbiological studies in the past decade. Among various technologies, mass spectrometry (MS)-based metabolomics has emerged as a key technology for the quantitative analysis of metabolites due to its extraordinary sensitivity, low sample consumption and high selectivity [10]. The approach has been recently applied to studies of various microalgae, including metabolite profiling of *C. reinhardtii* under nutrient deprivation [11], assessing the action mode of mixotrophic metabolism in the model diatom *Phaeodactylum tricornutum* [12], comparative metabolomic analysis of the green microalga *Chlorella sorokiniana* cultivated in the single culture and a consortium with bacteria for wastewater remediation [13], identification of trehalose as an abundant and diurnally fluctuating metabolite in the microalga *Ostreococcus tauri* [10], and dynamics metabolites during the cell cycle of *C. reinhardtii* [14]. Overall these results demonstrated that metabolomics could be a valuable tool in analyzing cell metabolism and the cellular responses to environmental factors.

Increasing the total lipid yield of microalgae is a major bottleneck in microalgae biofuel development. Currently few studies have addressed the addition of organic carbon source during nitrogen or phosphorus depletion as a potential means to increase both biomass and FA contents in microalgae [15]. The metabolic mechanism of microalgae FA accumulation and biomass under above treatments is also elusive. In this study, we used *C. reinhardtii* as the experimental model. We combined the cultivation conditions of nitrogen depletion and different phosphorus concentrations with the addition of sodium acetate as the organic carbon source, to increase lipid content and biomass simultaneously. In addition, through the analysis of total metabolites, we provide mechanistic insights into the regulation of lipid accumulation in microalgae.

## Methods

### Material and culture conditions

The green microalga *Chlamydomonas reinhardtii* CC124 was obtained from the *Chlamydomonas* Genetic Center of Duke University (Durham, NC, USA). Cells were cultured in Tris–Acetate–Phosphate (TAP) medium [16] at 25 ^***◦***^C and under continuous cool-white fluorescent lamps (≈ 100 μmol photons/m^2^ s) within a 110 rpm shaking incubator. Initially 5 × 10^5^ cells/mL was incubated with 100 mL medium in 150 mL Erlenmeyer flasks.

### Effects of phosphorus on the growth and lipid accumulation under nitrogen deficiency

After 3 days cultivation as described above, 100 mL cells (~ 2–4 × 10^6^ cells/mL) were pelleted and suspended in standard TAP medium, TAP nitrogen deficiency (T-N), TAP without nitrogen and phosphorus (T-N-P), TAP nitrogen deficiency with additional phosphorus 1 M(K)PO_4_ (T-N+P), each in triplicates (Table 1). The effects of nitrogen deficiency TAP medium with different phosphorus concentrations on the growth and lipid accumulation of *Chlamydomonas* were then studied. After media change, 1 mL of cells were collected every day for the growth curve, and total 10^7^ cells were retrieved at 3, 4, 5, and 6 days for GC–MS metabolomics, and the rest cells were harvested at the end of the treatments at 7 days for biomass and FA content determination. In this study, treatment with the highest lipid content, T-N-P, was selected to carry out the following next experiments in “Effects of acetate on growth, biomass and lipids in *Chlamydomonas*” section.

**Table 1:** The basic components of different treatments

Media	Composition
TAP^a^	Tris (2.42 g, 4× Beijerinck salts (25 mL), 1 M(K)PO_4_ (0.375 mL) Trace (1 mL), acetate acids (1 mL), ddH_2_O (975 mL)
T-N	NH_4_Cl in 4× Beijerinck salts change to KCl
T-N-P	NH_4_Cl in 4× Beijerinck salts change to KCl, without adding 1 M(K)PO_4_
T-N+P	NH_4_Cl in 4× Beijerinck salts change to KCl, adding two times 1 M(K)PO_4_

^a^Stock solutions for 1 L of TAP media: 1 M Tris base (e.g., Trizma) 20 mL, phosphate buffer II 1.0 mL solution A 10.0 mL, Hutner’s trace elements 1.0 mL, glacial acetic acid 1.0 mL, phosphate buffer II (for 100 mL) with K_2_HPO_4_ 10.8 g, KH_2_PO_4_ 5.6 g, solution A (for 500 mL) NH_4_Cl 20 g, MgSO_4_–7H_2_O 5 g, CaCl_2_–2H_2_O 2.5 g (adjust final pH to 7.0) [15]

### Effects of acetate on growth, biomass and lipids in *Chlamydomonas*

Similarly, cells from “Material and culture conditions” in “Methods” section were collected, washed and the medium was changed, respectively, on 3-day (~ 2–4 × 10^6^ cells/mL). Cells in T-N-P were spiked with 1 g/L of sodium acetate (T-N-P+1Ac), or 2 g/L of (T-N-P+2Ac), or 4 g/L sodium acetate (T-N-P+4Ac) under culture conditions as described in “Material and culture conditions” section. Similarly, cells were collected after media change for the growth curve, GC–MS metabolomics, biomass, and FA determination at the same time intervals as in “Effects of phosphorus on the growth and lipid accumulation under nitrogen deficiency” section.

### Contents and composition of fatty acid

Total lipid extraction was performed as described previously with slight modifications [8, 17]. Briefly, 5 mg lyophilized cells were suspended in 1 mL 2 M NaOH–CH_3_OH solution and shaken (110 rpm) for 1 h at room temperature (RT) and incubated at 75 ^***◦***^C for 15 min. After cooling down, the mixture was spiked with 1 mL 4 M HCl–CH_3_OH and pH was adjusted to below 2.0 with HCl, followed by incubation at 75 °C for 15 min. After that, FA methyl esters (FAMEs) were extracted with 1 mL hexane, shaking by hand for 30 s and then centrifuged at 3500*g* for 2 min. The hexane phase was collected and blow-dried, then 500 mL dichloromethane was added and samples were stored at − 20 ^***◦***^C for further GC–MS analysis. 50 μL of C19 (5 mg non-adecanoic acid-methyl ester dissolved in 10 mL dichloromethane) was added before extraction to estimate the recovery rate.

Qualification and quantification of FAMEs were performed on GC–MS (7890A-5975C, Agilent, USA) which was equipped with a HP-5MS column (30 m × 0.25-mm id, film thickness 0.25 μm). The temperature of the injector was maintained at 250 ^***◦***^C and the transmission line was maintained at 290 ^***◦***^C. Helium was used as the carrier gas and ions were generated by a 70 eV electron beam and the mass range scanned was 50–550 *m*/*z*. The oven temperature for FAME analysis was initially maintained at 70 ^***◦***^C for 4 min followed by a temperature rising rate of 25 ^***◦***^C/min to 195 ^***◦***^C, then held for 5 min, and then raised at 3 ^***◦***^C/min to 205 ^***◦***^C. Peak identification was performed by matching the mass spectra of each compound with the National Institute of Standards and Technology mass spectral library (NIST, 2005, Gaithersburg, MD). The datasets of FAME profiling for further analysis were obtained by normalization with internal standards in the same chromatograms [17, 18].

### GC–MS based metabolomics analysis

Metabolomic extraction was performed as described previously [19]. All chemicals used for metabolome isolation and GC–MS analyses were obtained from Sigma-Aldrich (Taufkirchen, Germany). After the cells were collected by centrifugation at 8000*g* for 3 min at 4 ^***◦***^C (Eppendorf 5430R, Hamburg, Germany), the algae pellets were immediately frozen in liquid nitrogen and then stored at – 80 ^***◦***^C before use. The metabolomic analysis protocol included: (i) Metabolome extraction: cells were re-suspended in 1 mL cold 10:3:1 (v/v/v) methanol: chloroform: H_2_O solution (MCW), and frozen in liquid nitrogen and thawed five times. Supernatants were collected by centrifugation at 15,000*g* for 3 min at 4 ^***◦***^C (Eppendorf 5430R, Hamburg, Germany). To normalize variations across samples, an internal standard (IS) solution was added to 200 μL supernatant in a 1.5-mL microtube before it was dried by vacuum centrifugation for 2–3 h (4 ^***◦***^C). (ii) Sample derivatization: derivatization was conducted according to the two-stage technique by Roessner et al. [20]. The samples were dissolved in 20 μL methoxyamine hydrochloride (20 mg/mL in pyridine) and shaken for 90 min at 30 ^***◦***^C. 90 μL *N*-methyl-*N*-(trimethylsilyl) trifluoroacetamide (MSTFA) was then added and samples were incubated at 37 ^***◦***^C for 30 min to trimethylsilylate the polar functional groups. The derived samples were collected by centrifugation at 15,000×*g* for 3 min before GC/MS analysis. (iii) GC–MS analysis: analysis was performed on a GC–MS system-GC 7890A coupled to an MSD 5975C (Agilent Technologies, Inc., Santa Clara, CA, USA) equipped with a HP-5MS capillary column (30 m × 250 mm id). 2 μL derived sample was injected in splitless mode at 230 ^***◦***^C injector temperature. Transmission line was maintained at 290 ^***◦***^C. The GC was operated at constant flow of 1 mL/min helium. The temperature program started in isocratic mode at 45 ^***◦***^C for 2 min, followed by temperature ramping of 5 ^***◦***^C/min to a final temperature of 280 ^***◦***^C, which was then held constantly for an additional 2 min. The range of mass scan was *m*/*z* 50–550. Peak identification was performed by matching the mass spectra of each compound with NIST. Automatic peak deconvolution was conducted with Masslynx software (Version 4.1, Waters Corp.). The metabolite profiles for further analysis were obtained by normalizing with the internal standards in the same chromatograms.

## Results and discussion

### Growth inhibition in nitrogen deficiency and different phosphorus concentrations

The present study aimed to determine the optimum growth conditions for lipid accumulation in *C. reinhardtii*. To do this we cultured *C. reinhardtii* in various culture conditions including TAP, T-N, T-N-P and T-N+P.

**Fig. 1:**
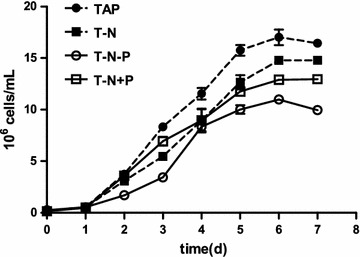
The growth curve of *C. reinhardtii* under different cultivation conditions, including with TAP, T-N, T-N-P and T-N+P medium, respectively

**Fig. 2:**
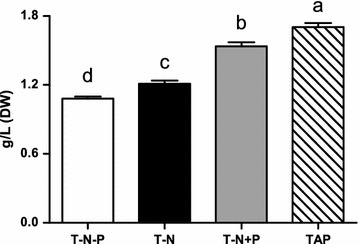
The dry weight biomass of *C. reinhardtii* under different cultivation conditions, including with TAP, T-N, T-N-P and T-N+P medium, respectively (Different letters on the top of the bar indicates they were significantly different at P ≤ 0.05 according to one-way ANOVA)

The fastest growth was observed in samples grown in standard TAP media, followed by T-N and T-N+P, and the slowest growth in T-N-P (Fig. 1). Similarly the highest biomass was obtained in TAP, following by T-N+P and T-N, with T-N-P having the lowest (Fig. 2). It seems that nitrogen deficiency and phosphorus deficiency both inhibit the division and growth of microalgal cells as nitrogen and phosphorus both are essential elements in cells. Under the condition of nitrogen deficiency, the microalgae growth was still inhibited even after phosphorus was added. This is in agree with the previous study conducted in another green microalga *Chlorella* where the initial nitrogen concentration was the major nutrient factor of growth and this was unaffected by initial phosphorus concentrations [20–22]. Manipulating environmental stresses and stress tolerance of microalgae is widely used to enhance production of lipids and value-added products [23]. The nutrient depletion is one of the most effective induction factors to trigger and enhance the lipid accumulation in microalgae [i.e., 23–26]. Among them, nitrogen-starvation is considered as an important and practically feasible way by which the lipid content can be enriched in large scale production [25]. The dilemma for lipid productivity in green microalgae: Microalgae grown under optimal conditions produce large amounts of biomass but with low neutral lipid content, while microalgae grown in nutrient starvation accumulate high levels of neutral lipids, but are slow growing [27]. It was reported that strains with a high capacity for both lipid accumulation and high photosynthetic activity under N starvation exhibited high lipid productivity over time [24]. Thus, there is a need for metabolic engineering of microalgae to constitutively produce high amounts of lipids without sacrificing growth.

### Increased lipid yield in nitrogen deficiency and different phosphorus concentrations

Next we used GC–MS to analyze the FA contents in *Chlamydomonas*, with nitrogen deficiency and in different phosphorus concentrations (Table 2). In all, 16 FA were detected, of which seven: C16: 0, C17: 0, C18: 0, C18: 1n9t, C18: 1n9c, C18: 2n6c and C18: 3n6 made up 90% of the total FA content (Table 2). The highest content of these FA was C16: 0, more than 37%, followed by C18: 3n6 with 25%. Saturated FA (SFA) and unsaturated FA (UFA) accounted for 45.3% and 54.7% of total FA (TFA), respectively, in TAP controls. In the treated samples, the percentage of SFA increased to more than 50%, in T-N and T-N+P samples, while decreased slightly in T-N-P samples. Previous studies also showed that the concentrations of nitrogen in the culture altered not only the TFA contents, but also the composition of TFA in microalgae [28].

**Table 2:** FA content (μg/mg) and TFA yield (mg) of *C. reinhardtii* under different conditions

	Treatments	TAP	T-N	T-N-P	T-N+P
FA content	C14:0	0.32 ± 0.04	0.36 ± 0.05	0.42 ± 0.02	0.44 ± 0.02
	C16:0	18.89 ± 0.16	30.61 ± 0.53	35.86 ± 0.25	34.04 ± 0.31
	C16:1	2.35 ± 0.17	1.52 ± 0.03	2.21 ± 0.05	1.91 ± 0.11
	C17:0	0.92 ± 0.17	3.88 ± 0.44	3.79 ± 0.30	4.18 ± 0.32
	C17:1	0.45 ± 0.11	0.46 ± 0.04	0.58 ± 0.04	0.56 ± 0.04
	C18:0	2.29 ± 0.21	3.44 ± 0.38	2.92 ± 0.11	3.70 ± 0.28
	C18:1n9t	2.38 ± 0.08	3.32 ± 0.18	3.91 ± 0.12	3.43 ± 0.05
	C18:1n9c	5.16 ± 0.12	7.18 ± 0.72	8.09 ± 0.07	8.28 ± 0.15
	C18:2n6c	3.83 ± 0.73	7.11 ± 0.67	22.12 ± 0.12	8.22 ± 0.31
	C18:3n3	0.08 ± 0.00	0.08 ± 0.00	0.05 ± 0.00	0.08 ± 0.00
	C18:3n6	12.94 ± 0.23	16.99 ± 0.29	22.84 ± 0.08	18.19 ± 0.38
	C20:0	0.66 ± 0.10	0.88 ± 0.08	1.09 ± 0.08	0.94 ± 0.06
	C20:1	0.60 ± 0.11	0.65 ± 0.14	0.77 ± 0.07	0.71 ± 0.02
	C20:2	0.13 ± 0.00	0.13 ± 0.00	0.12 ± 0.01	0.13 ± 0.00
	C21:0	0.14 ± 0.01	0.15 ± 0.02	0.12 ± 0.02	0.16 ± 0.00
	C22:6n3	0.16 ± 0.00	0.17 ± 0.00	0.09 ± 0.00	0.17 ± 0.00
	SFA	23.22 ± 0.51^c^	39.32 ± 1.38^b^	44.20 ± 0.08^a^	43.46 ± 0.82^a^
	MUFA	10.93 ± 0.33^c^	13.13 ± 1.07^b^	15.56 ± 0.30^a^	14.89 ± 0.06^a^
	PUFA	17.15 ± 0.51^d^	24.47 ± 0.96^c^	45.23 ± 0.19^a^	26.79 ± 0.65^b^
	TFA	51.30 ± 1.34^d^	76.92 ± 2.91^c^	105.00 ± 0.46^a^	85.15 ± 0.54^b^
	TFA yield (mg/L)	87.37 ± 1.21^d^	92.95 ± 2.21^c^	113.46 ± 1.78^b^	130.77 ± 3.78^a^

Different letters of each line in the superscript position indicates they were significantly different at P ≤ 0.05 according to one-way ANOVA

The highest TFA content was detected in T-N-P with 105.00 μg/mg followed by T-N+P and T-N, which were 85.15 and 76.92 μg/mg, respectively. Only 51.30 μg/mg TFA was detected in the TAP control samples (Table 2). Compared with the control group, TFA in T-N-P, T-N+P and T-N was increased by 104.7, 66.0 and 49.9%, respectively. Researchers reported that the TFA contents of microalgae increased under nitrogen and phosphorus deficiency conditions, especially in the presence of 0.04, 0.03 and 1 g/L of nitrate, phosphate, and sodium thiosulphate, respectively [29]. Improved TFA content was also observed in *Chlamydomonas* cultivated with T-N [30]. This showed that nitrogen deficiency and phosphorus deficiency together (T-N-P) significantly improved TFA accumulation in *Chlamydomonas* cells. Next we analyzed TFA yield under different treatments. TFA yield was calculated by the total FA contents and the DW biomass. We found the highest TFA yield in treatment of T-N+P with 130.77 mg/L followed by T-N-P and T-N, which were 113.46 and 92.95 mg/L, the lowest in TAP control was 87.37 mg/L (Table 2). Compared with the control group, TFA yield in T-N+P, T-N-P and T-N increased by 49.7, 29.9 and 6.4%, respectively. It seems that nitrogen deficiency increased TFA content per cell, but this occurred at the expense of biomass. This is consistent with previous results like in *Nannochloropsis oculata* and *Chlorella vulgaris* [6, 31–33].

The additional phosphorus reduced the TFA content per cell, however, with higher biomass as shown in Fig. 2 more TFA yield were obtained in this treatment in *Chlamydomanas*. That’s quite interesting without clear clue for the mechanism. Recent results showed that low phosphorus conditions increased both lipid content and lipid yield, however, it was also found that supplementing the growth media with K_2_HPO_4_ decreased lipid accumulation in cells of *Chlorella* sp. The analysis indicated that the carbohydrate content was directly correlated to phosphorus concentration [22]. Different nitrogen/phosphorus ratios in *Scenedesmus* sp. LX1 could differentially accumulate lipids but could not enhance the lipid yield under the conditions of higher lipid contents [31]. The lipid contents of *C. zofingiensis* grown in media deficient of nitrogen (65.1%) or phosphate (44.7%) were both higher than that obtained from cells grown in full medium (33.5%), which indicating nitrogen deficiency was more effective than phosphate deficiency for inducing lipid accumulation in *C. zofingiensis* [32]. Further research should be made on exactly what regulation under phosphorus addition to biomass and lipid accumulation and on how to enhance both lipid content and lipid yield in microalgae.

### Sodium acetate increases cell growth and lipid yield under condition of both nitrogen and phosphorus deficiency

**Fig. 3:**
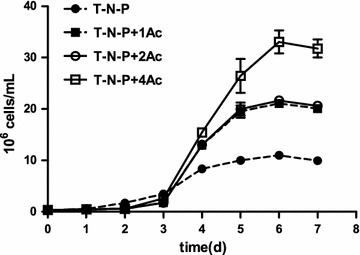
The growth curve of *C. reinhardtii* with different sodium acetate concentrations, as 1 g/L of sodium acetate (T-N-P+1Ac), or 2 g/L of (T-N-P+2Ac), or 4 g/L sodium acetate (T-N-P+4Ac)

To find the conditions that produce both high biomass and high TFA yield, we next tested the effects of different concentrations of sodium acetate as the carbon resource in combination with the absence of nitrogen and phosphorus. In our study, sodium acetate promoted the *Chlamydomonas* cell growth and increased biomass in a dose-dependent manner (Fig. 3). By day 7, cultures in T-N-P+1Ac and T-N-P+2Ac showed more than doubled and in T-N-P+4Ac more than tripled cell numbers (Fig. 3).

**Fig. 4:**
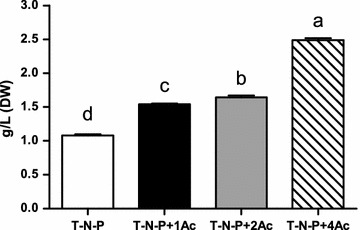
The dry weight biomass of *C. reinhardtii* with different sodium acetate concentrations, as 1 g/L of sodium acetate (T-N-P+1Ac), or 2 g/L of (T-N-P+2Ac), or 4 g/L sodium acetate (T-N-P+4Ac). (Different letters on the top of the bar indicates they were significantly different at P ≤ 0.05 according to one-way ANOVA)

Similarly, the addition of sodium acetate made the biomass increased significantly (Fig. 4). For example, the DW biomass of T-N-P treatment was 1.08 g/L, whereas the production in samples from T-N-P+1Ac, T-N-P+2Ac, and T-N-P+4Ac was 1.54, 1.64 and 2.49 g/L, respectively, i.e., 43, 52 and 130% higher than that of the T-N-P group. In conclusion, the addition of sodium acetate significantly increased cell number and biomass of *Chlamydomonas*, which is consistent with the previous reports in *C. pyrenoidosa*, with a certain amount of sodium acetate that can significantly promote the growth of microalgae [9].

The TFA compositions of nitrogen-deficient *Chlamydomonas* were unchanged by the addition of different concentrations of sodium acetate, with a total of 16 FA detected, including mainly the same types of FA as described in “Increased lipid yield in nitrogen deficiency and different phosphorus concentrations” section (Table 3). Next we analyzed the TFA production per mg in conditions of nitrogen and phosphorous depletion after the addition of different concentrations of sodium acetate. The highest TFA content was detected in T-N-P+1Ac, 120.45 μg/mg, followed by the T-N-P+2Ac and control treatment, which were 116.48 and 105.00 μg/mg, respectively, while the lowest TFA content was T-N-P+4Ac treatment with only 88.03 μg/mg (Table 3). We proposed that the addition of low concentration of sodium acetate (i.e., 1 g/L) under the condition of nitrogen and phosphorus deficiency could increase the TFA content, but TFA decreased with the increase of sodium acetate concentration no less than 2 g/L. Previous reports showed that TFA content increased in the absence of nitrogen and the addition of salt in *Chlamydomonas* [33]. Another study reported that a long-term supplementation of glucose with reduced supply of nitrogen in *Chlorella kessleri* allowed cells to accumulate lipids [34] We further analyzed the TFA yields under these treatments. In T-N-P the TFA yield was 113.46 mg/L, and the TFA yield increased with increasing sodium acetate concentrations (Table 3). For instance, TFA yield under T-N-P+1Ac and T-N-P+2Ac treatments was both around 190 g/L, while the highest TFA level was detected in the T-N-P+4Ac group, where it reached 218.98 g/L, i.e., 93.0% higher than that of the T-N-P control group. Our study therefore demonstrates that the addition of sodium acetate in the absence of nitrogen and phosphorous increases the TFA yield of *Chlamydomonas*. In a previous study, the maximum biomass and maximum lipid content were obtained when C, N, P at concentrations of 26.37, 2.61 and 0.03 g/L, respectively. All these suggested that these three elements (C, N, and P) were responsible for cell growth and lipid accumulation in microalgae [35, 36] and in this study we re-emphasize the importance of the ratio of the elements to lipid accumulation and biomass. What’s more, these results of metabolic and gene expression suggested that turnover of nitrogen-rich compounds such as proteins may provide carbon/energy for triacylglycerol (TAG) biosynthesis in the nutrient deprived cells in photoautotrophically grown microalgae *C*. *reinhardtii* and *Coccomyxa* sp*. C*-*169* [37]. Previous results also suggested that carbon availability is a key metabolic factor controlling oil biosynthesis and carbon partitioning between starch and oil in *Chlamydomonas*. In WT oil accumulation under T-N was strictly dependent on the available external acetate supply and the amount of oil increased steadily as the acetate concentration increased to the levels several-fold higher than that of the TAP [15]. What’s more, another study clearly demonstrated that inorganic carbon availability represent one of limiting factors for lipid and TAG accumulation under nutrient-depleted marine haptophyte *Pavlova lutheri* cultures [38]. Other report showed that the selection of the appropriate cultivation conditions is the key to improve lipid accumulation under nitrogen depletion by the unicellular green microalga *Scenedesmus obliquus* [39]. According to a recent study, both photosynthetic incorporation of inorganic carbon and the maximum rate of O_2_ evolution in *C. reinhardtii* can be significantly diminished by growth in the presence of acetate [40]. The direct addition of carbon may further enhance the TAG biosynthesis by providing building materials in this study. Further studies focusing on the regulation mechanisms behind the carbon supplement against the lipid accumulation would be of great help for the manipulation of microalgal bioenergetic strategy.

**Table 3:** FA content (μg/mg) and TFA yield (mg) of *C. reinhardtii* in different sodium acetate concentrations

	Treatments	T-N-P	T-N-P+1Ac	T-N-P+2Ac	T-N-P+4Ac
FA content	C14:0	0.42 ± 0.02	0.40 ± 0.00	0.37 ± 0.06	0.34 ± 0.04
	C16:0	35.86 ± 0.25	40.41 ± 3.11	42.45 ± 0.40	32.89 ± 0.25
	C16:1	2.21 ± 0.05	2.65 ± 0.30	2.55 ± 0.10	2.62 ± 0.19
	C17:0	3.79 ± 0.30	4.14 ± 0.04	3.58 ± 0.06	3.72 ± 0.33
	C17:1	0.58 ± 0.04	0.56 ± 0.05	0.58 ± 0.04	0.25 ± 0.01
	C18:0	2.92 ± 0.11	3.63 ± 0.04	3.13 ± 0.76	3.26 ± 0.29
	C18:1n9t	3.91 ± 0.12	7.42 ± 0.48	7.44 ± 0.54	8.29 ± 0.31
	C18:1n9c	8.09 ± 0.07	8.85 ± 0.49	7.05 ± 0.19	6.58 ± 0.21
	C18:2n6c	22.12 ± 0.12	28.92 ± 0.52	27.37 ± 0.39	16.51 ± 0.16
	C18:3n3	0.05 ± 0.00	1.02 ± 0.01	0.01 ± 0.00	0.01 ± 0.00
	C18:3n6	22.84 ± 0.08	21.53 ± 0.39	20.28 ± 0.37	12.24 ± 0.12
	C20:0	1.09 ± 0.08	0.71 ± 0.01	0.93 ± 0.05	0.57 ± 0.05
	C20:1	0.77 ± 0.07	0.09 ± 0.00	0.56 ± 0.09	0.55 ± 0.08
	C20:2	0.12 ± 0.01	0.07 ± 0.00	0.06 ± 0.02	0.05 ± 0.03
	C21:0	0.12 ± 0.02	0.01 ± 0.01	0.09 ± 0.02	0.10 ± 0.01
	C22:6n3	0.09 ± 0.00	0.04 ± 0.01	0.03 ± 0.00	0.03 ± 0.01
	SFA	44.20 ± 0.08^b^	49.3 ± 3.18^a^	50.55 ± 0.79^a^	40.88 ± 0.76^c^
	MUFA	15.56 ± 0.30^b^	19.57 ± 1.21^a^	18.18 ± 0.80^a^	18.31 ± 0.38^a^
	PUFA	45.23 ± 0.19^c^	51.58 ± 0.89^a^	47.75 ± 0.66^b^	28.84 ± 0.26^d^
	TFA	105.00 ± 0.46^b^	120.45 ± 5.07^a^	116.48 ± 1.07^a^	88.03 ± 0.35^c^
	TFA yield (mg/L)	113.46 ± 1.78^c^	185.61 ± 7.40^b^	191.55 ± 1.60^b^	218.98 ± 2.02^a^

Different letters of each line in the superscript position indicates they were significantly different at P ≤ 0.05 according to one-way ANOVA

Another strategy to improve the biomass and lipid production may be the addition of other chemicals, such as metal ions like Fe^3+^, Mg^2+^ and Ca^2+^ and EDTA [41]. It exhibited increasing the concentrations of metal ions can be beneficial to lipid accumulation of heterotrophic *Scenedesmus* sp. cells [41]. Together with addition of carbon source, metal ions and EDTA, we may find a potential solution to simultaneously enhance both biomass and lipid accumulation in microalgae.

### Metabolomics analyses of *Chlamydomonas* under nutrient deprivation

A total 72 metabolites appearing in all samples were detected by GC–MS (Additional file 1: Table S1, Additional file 2: Table S2). A similar study about the metabolic pathways of *Chlamydomonas* under the conditions of nitrogen deficiency, phosphorus deficiency and sulfur deficiency identified about 100 metabolites, including amide and amines related to nitrogen metabolism [42]. A similar number of metabolites were also reported in the study of *Chlamydomonas* and the changes in key amino acids and enzymes were analyzed [11].

**Fig. 5:**
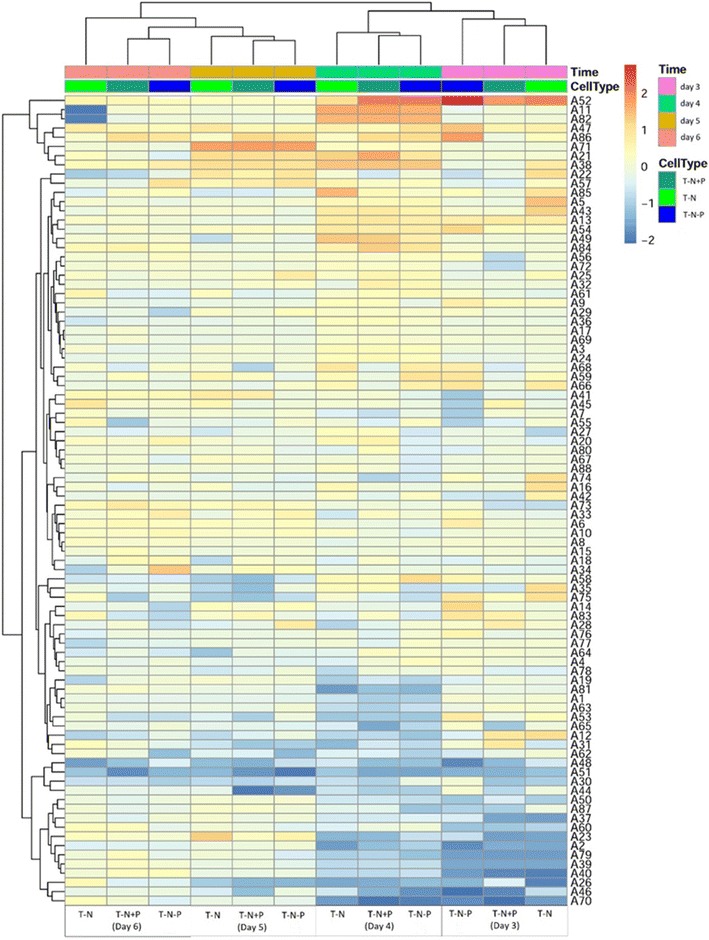
The metabolic heat map of *C. reinhardtii* under different cultivation conditions, including with T-N, T-N-P and T-N+P medium, respectively

**Fig. 6:**
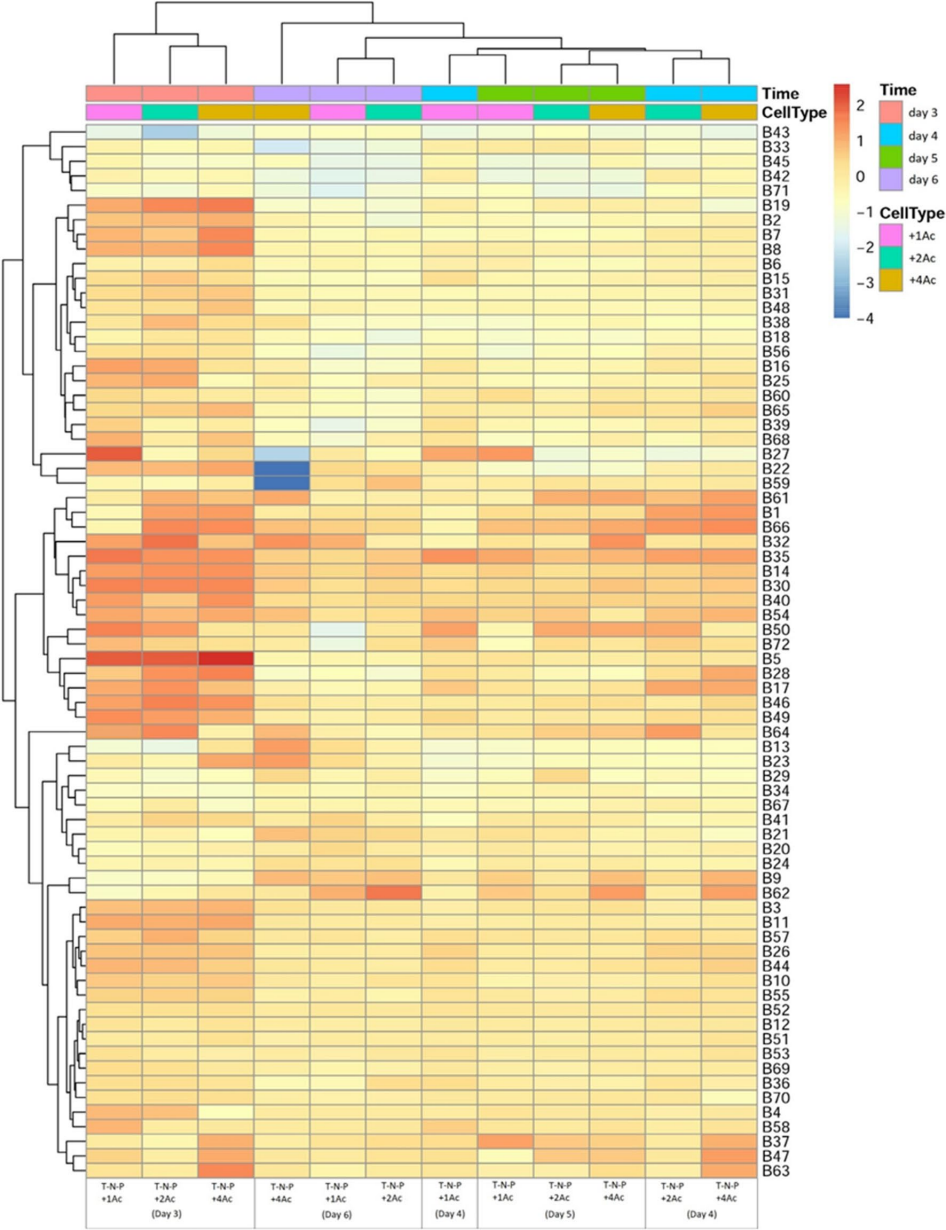
The metabolic heat map of *C. reinhardtii* with different sodium acetate concentrations, as 1 g/L of sodium acetate (T-N-P+1Ac), or 2 g/L of (T-N-P+2Ac), or 4 g/L sodium acetate (T-N-P+4Ac)

Next we plotted a heatmap using metabolomics data under different phosphorus concentrations in *Chlamydomonas* with nitrogen deficiency (Fig. 5). As expected, the stress of nutrient deficiency reduced the metabolomic level and intracellular metabolic capacity. Furthermore some metabolites were altered by nitrogen deficiency but no obvious changes were observed in different phosphorous concentrations. For instance, nitrogen deprivation decreased metabolites such as alpha-linolenic acid, acetic acid, propanoic acid and naphthalene but increased glycine, glycerol, hexadecanoic acid and phosphate. Glycerol, hexadecanoic acid, and phosphate are the end-products of metabolic pathways that may promote the lipid synthesis and accumulation, which is consistent with our observation that nitrogen deprivation increase TFA content. Metabolome analysis revealed betaine lipids as major source for triglyceride formation, and the accumulation of sedoheptulose during nitrogen-starvation, resulting in an increase in neutral lipids during nitrogen-depletion and predominantly 16:0 and 16:1 (*n*-7) accumulated in the TAG fraction in *P*. *tricornutum* [43]. Then we analyzed *Chlamydomonas* metabolomics upon the addition of sodium acetate. Sodium acetate addition increased the contents of hexadecanoic acid, alpha-linolenic acid, propanoic acid, phosphate, glycine, myristic acid and 1,2-benzenedicarboxylic acid (Fig. 6). Sodium acetate also increased whole metabolomic levels, which may significantly promote the growth of microalgae. Although sodium acetate reduced the TFA production (per mg dried weight), it increased the biomass significantly. Therefore, more lipids were produced upon the addition of sodium acetate in nitrogen-deficient conditions. A work in marine microalga *Chlamydomonas* sp. JSC4 demonstrated the synergistic integration of cultivation and dynamic metabolic profiling technologies to develop a simple and effective strategy for enhancing oil production [44]. The microalgae metabolite profiling appears to be a well suited method to detect numerous changes of metabolite levels in response to environmental stimuli and it will be useful to derive specific hypotheses of how metabolic activity adjusts in response to external stimuli and also for investigation of microalgae biofuels and biomass, as described in the previous study [11].

## Conclusions

In this study we sought to ascertain the optimum conditions for biofuel production in *Chlamydomonas*. We found that TFA production per DW could be maximized by restricted access to both nitrogen and phosphorus and the addition of 4 g/L sodium acetate under the condition of nutrient deficiency could significantly increase both the biomass and TFA yield (by 93.0%). By the whole cell metabolomics, we identified several metabolites that may play an important role in this increased TFA production and increase microalgal biomass.

## Additional files

**Additional file 1: Table S1.** Annotated and classified metabolites detected in *C. reinhardtii* under different cultivation conditions, including with T-N, T-N-P and T-N+P medium respectively.

**Additional file 2: Table S2.** Annotated and classified metabolites detected in *C. reinhardtii* with different sodium acetate concentrations, as 1 g/L of sodium acetate (T-N-P+1Ac), or 2 g/L of (T-N-P+2Ac), or 4 g/L sodium acetate (T-N-P+4Ac).
