# Supplementary material for: Growth and lipid accumulation by different nutrients in the microalga Chlamydomonas reinhardtii
Source: Biotechnol Biofuels. 2018 Feb 13;11:40. doi: 10.1186/s13068-018-1041-z (PMC5809890; doi:10.1186/s13068-018-1041-z)
Supplement: Supplementary file 1 — Additional file 1: Table S1. Annotated and classified metabolites detected in C. reinhardtii under different cultivation conditions, including with T-N, T-N-P and T-N+P medium respectively. [file 13068_2018_1041_MOESM1_ESM.doc]

**Table S1** Annotated and classified metabolites detected in *C. reinhardtii* under the conditions of nitrogen deficiency and different phosphorus concentrations

| # | metabolite |  |
| --- | --- | --- |
| A1 | Octadecanenitrile |  |
| A2 | .alpha.-Linolenic acid |  |
| A3 | Octadecanoic acid |  |
| A4 | Pentadecanenitrile |  |
| A5 | phosphate |  |
| A6 | Tetradecanoic acid |  |
| A7 | Hexadecanamide |  |
| A8 | Tetradecanamide |  |
| A9 | Oleic acid |  |
| A10 | 9,12-Octadecadienoic acid (Z,Z)- |  |
| A11 | Hexadecanoic acid |  |
| A12 | Phosphoric acid |  |
| A13 | glycerol |  |
| A14 | Heptacosane |  |
| A15 | Octadecanoic acid，2,3-bis[(trimethylsilyl)oxy]propyl ester |  |
| A16 | Glucose oxime hexakis |  |
| A17 | Myo-Inositol, 1,2,3,4,5,6-hexakis-O |  |
| A18 | Octacosane |  |
| A19 | Octadecanamide |  |
| A20 | Propanoic acid |  |
| A21 | Glycine |  |
| A22 | Eicosane |  |
| A23 | Trisiloxane,1,1,1,5,5,5-hexamethyl-3,3- |  |
| A24 | Trisiloxane, octamethyl- |  |
| A25 | l-Mannopyranose, 6-deoxy-1,2,3,4-tetrakis-O-(trimethylsilyl)- |  |
| A26 | Cycloheptasiloxane, tetradecamethyl- |  |
| A27 | 2,6 ditert.-butylphenoxy |  |
| A28 | Tetradecanenitrile |  |
| A29 | Heptacosane |  |
| A30 | 2-Monopalmitin |  |
| A31 | Tridecane |  |
| A32 | Coronene, methyl- |  |
| A33 | 3,7-Dioxa-2,8-disilanonane, 2,2,8,8-tetramethyl- |  |
| A34 | 3,6-Dioxa-2,4,5,7-tetrasilaoctane,2,2,4,4,5,5,7,7-octamethyl- |  |
| A35 | Heneicosane |  |
| A36 | 4-Methylthio-N-phenyl-1,2-carbazoledicarboximide |  |
| A37 | Octadecane, 1-iodo- |  |
| A38 | Hexadecanoic acid, 2,3-bis[(trimethylsilyl)oxy]propyl ester |  |
| A39 | 6,7-Dihydroxy-5,8,13,14-pentaphenetetrone |  |
| A40 | Estra-1,3,5(10)-trien-16-one, 3-[(trimethylsilyl)oxy]- |  |
| A41 | Silane, [(1-methyl-1,3-propanediyl)bis(oxy)]bis[trimethyl- |  |
| A42 | 3-Bromo-5-ethoxy-4-hydroxybenzaldehyde |  |
| A43 | Benzenamine, 2-methyl-5-nitro-N-(4-methylthiobenzylidene)- |  |
| A44 | Octadecane |  |
| A45 | N,N-Dimethyl-4-(3-oxo-3-thiophen-2-yl-propylamino)-benzamide |  |
| A46 | Propanedioic acid |  |
| A47 | Hexacosane |  |
| A48 | Hexanedioic acid |  |
| A49 | 1H-Pyrrole, 2,3,4,5-tetraphenyl- |  |
| A50 | Cyclononasiloxane, octadecamethyl- |  |
| A51 | Tetracosane |  |
| A52 | Benzo[b]naphtho[2,1-d]thiophene |  |
| A53 | Acetic acid |  |
| A54 | Acetamide, N-(4-phenylbutyl)- |  |
| A55 | Pentacosane |  |
| A56 | D-Erythro-Pentofuranose, |  |
| A57 | Ethanimidic acid |  |
| A58 | 1,4-Butanediamine, N,N,N',N'-tetrakis(trimethylsilyl)- |  |
| A59 | 2,2-Dimethyl-1-pentamethyldisilanyloxypropane |  |
| A60 | 7-Dimethyl(trimethylsilyl)silyloxytetradecane |  |
| A61 | Silanol, trimethyl-, carbonate (2:1) |  |
| A62 | D-Mannitol, 1,2,3,4,5,6-hexakis-O-(trimethylsilyl)- |  |
| A63 | 3,8-Dioxa-2,9-disiladecane, 2,2,9,9-tetramethyl- |  |
| A64 | Disiloxane, 1,3-bis(1,1-dimethylethyl)-1,1,3,3-tetramethyl- |  |
| A65 | Pentadecane |  |
| A66 | Silanamine, N-[2-[3,4-bis[(trimethylsilyl)oxy]phenyl]ethyl]- |  |
| A67 | 3H,3'H,3"H-Trisindeno[1,2-a:2',1'-c:1",2"-e]benzene |  |
| A68 | Silanamine, 1,1,1-trimethyl-N-(trimethylsilyl)-N-[2-[(trimethylsilyl)oxy]ethyl]- |  |
| A69 | Pyridine, 3-trimethylsiloxy- |  |
| A70 | Docosane |  |
| A71 | 5-Oxazolecarboxamide, 2-phenyl- |  |
| A72 | Trisiloxane, octamethyl- |  |
| A73 | 1H-Indole, 4-methoxy- |  |
| A74 | Benzo[1,2-b:5,4-b']bisbenzofuran |  |
| A75 | p-(Dimethylamino)cinnamic acid |  |
| A76 | N,o,o'-Tris-(trimethylsilyl)tyrosine |  |
| A77 | Tetrasiloxane, decamethyl- |  |
| A78 | Benzene, 1,2-diethyl-3,4-dimethyl- |  |
| A79 | Hydantoin |  |
| A80 | Amiphenazole |  |
| A81 | Bicyclo[3.1.1]heptane |  |
| A82 | Lepidine, 8-nitro- |  |
| A83 | 1,4-Dimethylphenoxathin |  |
| A84 | Quinoline, 1,2,3,4-tetrahydro-2-methyl- |  |
| A85 | 1-Methyl-2-pentamethyldisilanyloxycyclohexane |  |
| A86 | 2-Pyrrolidino-1-(p-tolyl)-1-trimethylsilyloxyhexane |  |
| A87 | Naphthalene, 2-ethoxy- |  |
| A88 | Silane, 1,8-octanediylbis[trimethyl- |  |
