# Supplementary material for: Growth and lipid accumulation by different nutrients in the microalga Chlamydomonas reinhardtii
Source: Biotechnol Biofuels. 2018 Feb 13;11:40. doi: 10.1186/s13068-018-1041-z (PMC5809890; doi:10.1186/s13068-018-1041-z)
Supplement: Supplementary file 2 — Additional file 2: Table S2. Annotated and classified metabolites detected in C. reinhardtii with different sodium acetate concentrations, as 1 g/L of sodium acetate (T-N-P+1Ac), or 2 g/L of (T-N-P+2Ac), or 4 g/L sodium acetate (T-N-P+4Ac). [file 13068_2018_1041_MOESM2_ESM.doc]

**Table S2** Annotated and classified metabolites detected in *C. reinhardtii* in different sodium acetate concentrations, under the conditions of nitrogen and phosphorus deficiency

| # | metabolite |  |
| --- | --- | --- |
| B1 | Octadecanenitrile |  |
| B2 | .alpha.-Linolenic acid |  |
| B3 | Octadecanoic acid |  |
| B4 | Pentadecanenitrile |  |
| B5 | phosphate |  |
| B6 | Tetradecanoic acid |  |
| B7 | Hexadecanamide |  |
| B8 | Tetradecanamide |  |
| B9 | Oleic acid |  |
| B10 | 9,12-Octadecadienoic acid (Z,Z)- |  |
| B11 | Hexadecanoic acid |  |
| B12 | glycerol |  |
| B13 | Heptacosane |  |
| B14 | Octadecanoic acid |  |
| B15 | Octacosane |  |
| B16 | Octadecanamide |  |
| B17 | Propanoic acid |  |
| B18 | Glycine |  |
| B19 | Eicosane |  |
| B20 | Trisiloxane,1,1,1,5,5,5-hexamethyl-3,3- |  |
| B21 | Cycloheptasiloxane |  |
| B22 | Tetradecanenitrile |  |
| B23 | Heptacosane |  |
| B24 | 2-Monopalmitin |  |
| B25 | Tridecane |  |
| B26 | 3,7-Dioxa-2,8-disilanonane, 2,2,8,8-tetramethyl- |  |
| B27 | 3,6-Dioxa-2,4,5,7-tetrasilaoctane,2,2,4,4,5,5,7,7-octamethyl- |  |
| B28 | Heneicosane |  |
| B29 | Octadecane, 1-iodo- |  |
| B30 | Hexadecanoic acid, 2,3-bis[(trimethylsilyl)oxy]propyl ester |  |
| B31 | Estra-1,3,5(10)-trien-16-one, 3-[(trimethylsilyl)oxy]- |  |
| B32 | Silane, [(1-methyl-1,3-propanediyl)bis(oxy)]bis[trimethyl- |  |
| B33 | Octadecane |  |
| B34 | Hexacosane |  |
| B35 | 1H-Pyrrole, 2,3,4,5-tetraphenyl- |  |
| B36 | Cyclononasiloxane, octadecamethyl- |  |
| B37 | Tetracosane |  |
| B38 | Pentacosane |  |
| B39 | Ethanimidic acid |  |
| B40 | 1,4-Butanediamine, N,N,N',N'-tetrakis(trimethylsilyl)- |  |
| B41 | 7-Dimethyl(trimethylsilyl)silyloxytetradecane |  |
| B42 | Pentadecane |  |
| B43 | Silanamine,N-[2-[3,4bis[(trimethylsilyl)oxy]phenyl]ethyl]- |  |
| B44 | 3H,3'H,3"H-Trisindeno[1,2-a:2',1'-c:1",2"-e]benzene |  |
| B45 | Silanamine,1,1,1-trimethyl-N-(trimethylsilyl)-N-[2-[(trimethylsilyl)oxy]ethyl]- |  |
| B46 | Pyridine |  |
| B47 | Docosane |  |
| B48 | Tetrasiloxane, decamethyl- |  |
| B49 | 3-Heptadecene, (Z)- |  |
| B50 | Heptadecanenitrile |  |
| B51 | Oleanitrile |  |
| B52 | Phytol |  |
| B53 | Pentasiloxane, dodecamethyl- |  |
| B54 | 1,2-Benzenedicarboxylic acid, mono(2-ethylhexyl) ester |  |
| B55 | 1-(3-Methylbutyl)-2,3,4,6-tetramethylbenzene |  |
| B56 | 2-Amino-6-t-butyl-3-cyano-4,5,6,7-tetrahydrothianaphthene |  |
| B57 | N,N,O,O'-Tetrakis(trimethylsilyl)normetanephrine |  |
| B58 | n-Butylamine |  |
| B59 | Myristic acid |  |
| B60 | Benzyloxyamine |  |
| B61 | Picolinamide |  |
| B62 | 6-Ethyl-3-(dimethyl(prop-2-enyl)silyloxy)decane |  |
| B63 | Pyridinium, 1-(2-hydrazino-2-oxoethyl)-, chloride |  |
| B64 | Borneol, pentamethyldisilanyl ether |  |
| B65 | Hentriacontane |  |
| B66 | Dibenzothiophene, 5-oxide |  |
| B67 | Nonahexacontanoic acid |  |
| B68 | 5-Dimethyl(trimethylsilyl)silyloxytridecane |  |
| B69 | 2-Pentamethyldisilanyloxypentane |  |
| B70 | 1,1,3,3-Tetramethyl-1,3-di-n-propyldisiloxane |  |
| B71 | Chloropyrazine |  |
| B72 | Piperazine,1-ethoxycarbonyl-4-(2-fluorobenzoylaminothiocarbonyl)- |  |
